# The origins of giant viruses, virophages and their relatives in host genomes

**DOI:** 10.1186/s12915-014-0051-y

**Published:** 2014-06-30

**Authors:** Aris Katzourakis, Amr Aswad

**Affiliations:** 1 Department of Zoology, University of Oxford, Oxford OX1 3PS, UK

## Abstract

Giant viruses have revealed a number of surprises that challenge conventions on what constitutes a virus. The Samba virus newly isolated in Brazil expands the known distribution of giant mimiviruses to a near-global scale. These viruses, together with the transposon-related virophages that infect them, pose a number of questions about their evolutionary origins that need to be considered in the light of the complex entanglement between host, virus and virophage genomes.

See research article: http://www.virologyj.com/content/11/1/95.

## The discovery of giant viruses

Giant DNA viruses are double-stranded DNA (dsDNA) viruses that have particle and genome sizes comparable to those of small bacteria, and a number of features that are uncharacteristic of viruses. These include the presence of several genes that are similar to cellular genes such as those involved in DNA repair, translation, protein folding, and polysaccharide synthesis [[1]]. *Acanthamoeba polyphaga* mimivirus was the first of the giant DNA viruses to be discovered, initially isolated in the search for the causative agent of pneumonia during a hospital outbreak in Bradford, UK [[1]]. Since then, related viruses have been identified in a range of environments, including the discovery last year of the morphologically and genetically distinct pandoraviruses, which are even larger than the mimiviruses [[2]]. More recently, the 30,000-year-old *Pithovirus sibericum* was unearthed and brought back to life from Siberian permafrost [[3]].

Many of the giant viruses discovered to date have amoeba hosts and amoeba culture techniques have proved instrumental in identifying these giants, including the discovery last month of Samba virus, a wild mimivirus from the Amazonian Rio Negro [[4]]. Although slightly larger, Samba virus shares identity across the majority of its genome to the original Bradford mimivirus, further expanding the widespread distribution of these giant viruses. The defining feature of giant viruses is that they are an extreme outlier in terms of genome size: *Acanthamoeba polyphaga* mimivirus has a 1.2 Mb genome [[1]], which was double the size of the largest virus known at the time, and pandoravirus genomes reach up to 2.5 Mb [[2]]. Giant viruses are also extreme outliers in terms of their physical size, being too large to pass through porcelain filters, a criterion historically used to define a virus. As a further challenge to the traditional definition of viruses, giant viruses have several essential protein synthesis genes that have thus far been thought to be exclusive to cellular life [[1]].

## Evolutionary origins of viral giantry

Determining the evolutionary relationships among viruses is crucial to investigating the origins of features such as their size, but is complicated by the absence of universally conserved viral genes. The Baltimore system classifies viruses according to genome type and replication strategy, therefore placing giant viruses among others with dsDNA genomes. They are also considered on the basis of distinguishing biological features to belong within the nucleocytoplasmic large DNA viruses (NCLDVs) alongside viral families such as poxviruses and iridoviruses [[5]]. While dsDNA viruses in general do not appear to have a single evolutionary origin, the NCLDVs all contain five core genes and tend to share a suite of 50 or so likely ancestral genes [[5]] that partition them from other large eukaryotic dsDNA viruses such as nudiviruses, herpesviruses and baculoviruses. NCLDVs do share some genes with these other large DNA viruses, but are additionally distinguished by an either completely or largely cytoplasmic replication cycle [[5]].

**Figure 1:**
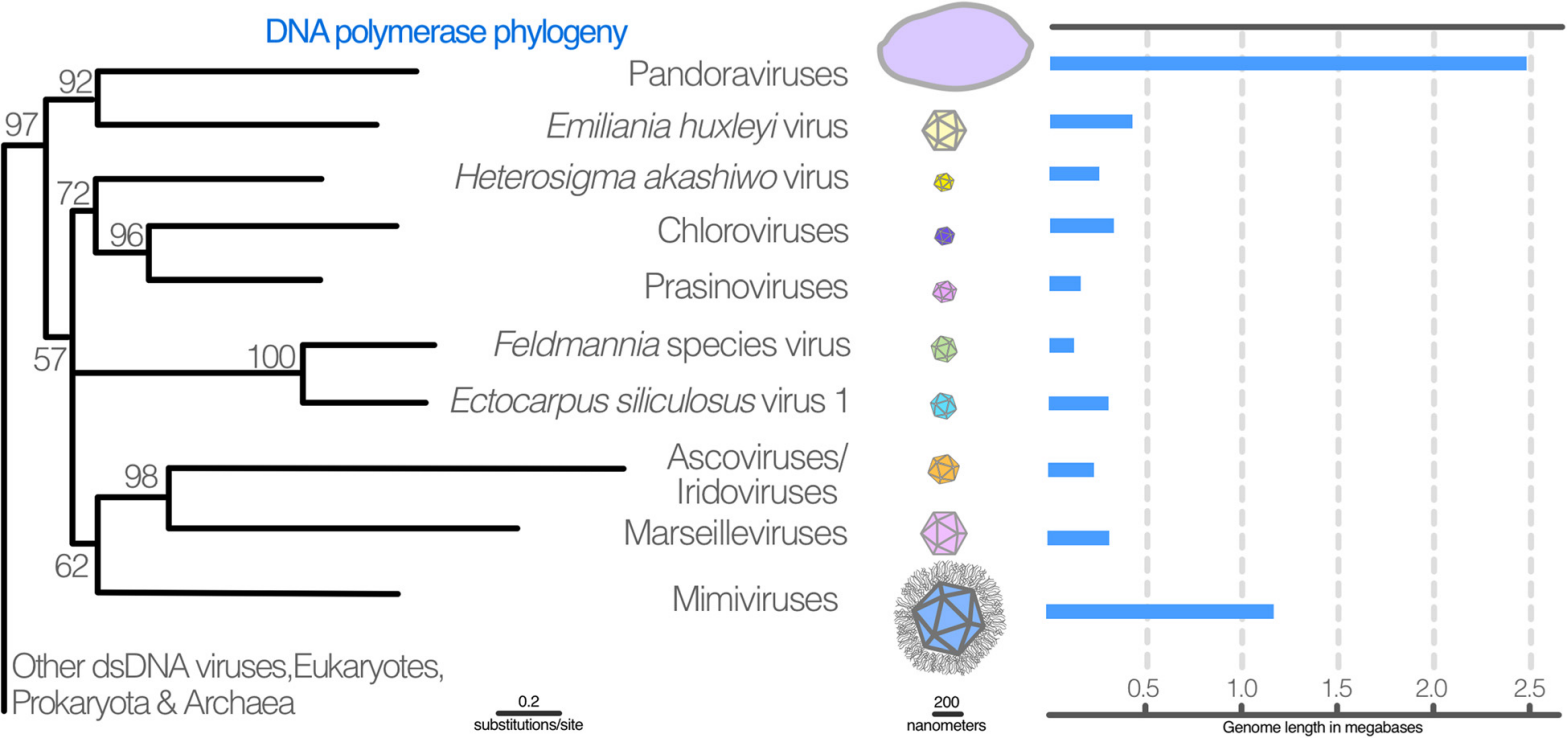
**Stylized figure depicting the phylogenetic relationships, genome length and virion size of various nucleocytoplasmic large DNA viruses (NCLDVs).** The maximum likelihood tree shown is a simplified version of the NCLDV subtree for DNA polymerase adapted from [[6]], where collapsed clades in the original tree are represented by single branches. Numbers at each node are expected likelihood weights from 1,000 rearrangements. The scale throughout the figure is approximate.

Although the low levels of genetic similarity among NCLDVs complicate the precise phylogenetic placement of giant viruses, the relationships between NCLDV families have been reconstructed using multiple conserved genes [[5]]. The phylogenetic relationships of DNA polymerase genes from NCLDVs reveal that the Mimiviridae family, one of the larger members of which is the Samba virus [[4]], groups with the Marseilleviridae and Iridoviridae that are 1.2 Mb, 350 kbp and 200 kbp in size, respectively (Figure 1). Pandoraviruses are most closely related to *Emiliana huxleyi* virus, which has a genome that is 0.41 Mb in size. Thus, pandoraviruses are derived members of Phycodnaviridae and therefore phylogenetically distinct from the rest of the giant viruses [[2],[5]]. While it has been argued that the large genomes of these giants suggest a large and complex ancestor, the most parsimonious interpretation of the phylogenetic evidence is that viral giantry evolved independently on at least two occasions from ancestors with much smaller genomes [[5]] (Figure 1).

The genome sizes of the NCLDVs vary greatly, from the 150 kb genomes of the poxviruses to the 2.5 Mb genomes of pandoraviruses [[2],[5]]. This hints at the possibility that viruses with intermediate genome sizes may exist. While many lineages may be extinct, it seems likely that at least some will eventually be found through metagenomic sampling. It would be premature to conclude that mimivirus and pandoravirus represent the largest DNA viruses that will be found. Interestingly, the recently uncovered pithoviruses are phylogenetically closer to the mimivirus/marseillevirus group despite a morphological resemblance to pandoraviruses that have ovoid rather than icosahedral morphology [[3]]. Moreover, of the more than 1,000 pandoravirus genes, 93% are previously unknown to biologists [[2]]. Many viruses contain some of these so-called orphan genes, but the high percentage of orphans in a single virus highlights how limited our sampling of the diversity of viral genes is.

## Giant viruses are susceptible to viral infection by relatives of eukaryotic DNA transposons

**Figure 2:**
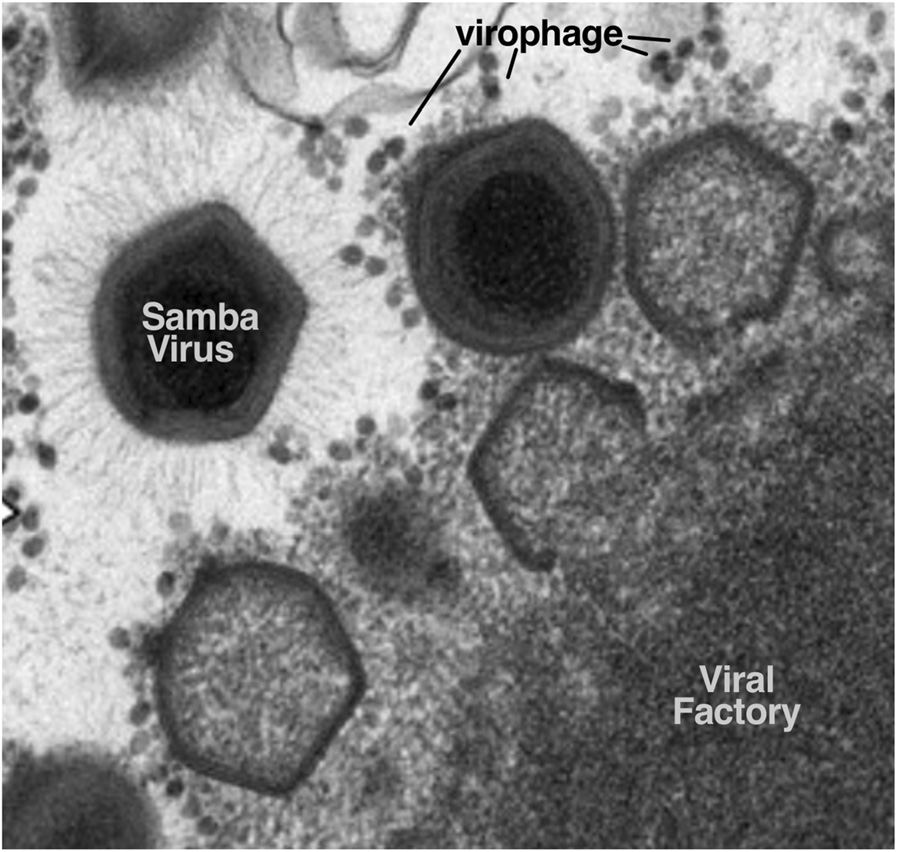
**Scanning electron microscope image of Samba virus and Rio Negro virophage adapted from[**[4]**].** The figure depicts Samba virus morphogenesis within the viral factory that is formed in *Acanthamoeba castellanii,* as well as the parasitic virophage particles interspersed among the giant virions.

Giant viruses reproduce in ‘viral factories’, which are cytoplasmic compartments of the host cell that can be as large as the nucleus (Figure 2). Accompanying the surprise of discovering giant viruses was the discovery of a group of associated viruses that are not capable of replication in their absence, and instead exploit the viral factory for replication. Other viruses are known to require helper viruses for replication, but these ‘virophages’ result in the formation of defective mimiviruses, implying a parasitic relationship [[6]]; they make the giant viruses ill. For example, the infection of Samba virus by its virophage results in a reduction of viral titer of over 80%, as well as partial recovery of the host amoebae [[4]]. This parasitism is therefore part of a complex relationship between giant viruses, their hosts and the virophages [[6]]. Virophage genomes are dwarfed by the mimiviruses that they infect, being approximately 20 kbp in length, and have been identified in association with several members of Mimiviridae [[6],[7]]. There are now multiple strains of the first virophage, named Sputnik [[6]], including the Rio Negro isolate from the Samba virus [[4]] and more distantly related virophages such as Mavirus, identified from the *Cafeteria roenbergensis* virus (CroV) [[7]].

Virophages are related to a class of eukaryotic DNA transposons called Mavericks (or Polintons). These genomic parasites share a set of four core genes [[7]], as well as widespread conservation of the characteristically viral jelly-roll capsid [[8]]. Two of these core genes are present in virophages, indicating a close evolutionary relationship, and the Mavirus virophage in particular shares a total of seven homologs with Mavericks [[7],[8]]. This indicates a much closer evolutionary relationship between Mavirus and Maverick transposons than Mavirus has with other virophages like Sputnik, with which it only shares four genes in total [[7]]. Mavericks are thought to derive from a DNA virus that integrated into the host genome, and the discovery of Mavirus strongly suggests a virophage-like progenitor to Mavericks. Several features indicate that the Mavirus ancestor was also a virophage rather than an escaped Maverick-like transposon [[7]], although this has been debated [[8]]. One such notable feature is the dependence on CroV for replication, as indicated by the high similarity of Mavirus promoters to those of CroV [[7]]. It is hard to imagine how a DNA transposon’s replication strategy would evolve to rely on CroV infection, whereas the post-integration loss of this feature in Mavericks is more readily explained [[7]]. Interestingly, some Maverick elements are more closely related to some virophages than to other Mavericks [[8]], suggesting that these integrations are a recurring event. The fact that Mavericks are widespread in the animal kingdom indicates that a number of virophages, and therefore NCLDVs, are yet to be discovered in association with these hosts.

## A question of gene flow and its evolutionary consequences

Gene flow has played a central role in the evolutionary history of virophages. Integrated virophages have been found in a mimivirus genome, and virophage genes also share similarity to genes in other DNA transposons, such as a class of linear plasmids called transpovirons that are also found in mimiviruses [[9]]. Some virophage genes also show similarity to bacteriophages, cellular genes, and their respective viral hosts [[7]]. This compound nature of virophage genomes is evidence of extensive horizontal gene transfer, and although the precise details of this gene flow are not fully understood, perspectives from paleovirology - the study of viral remnants, or ‘fossils’, found in host genomes - may help to clarify them. Analysis of these viral remnants, known as endogenous viral elements (EVEs), has revealed that all viruses can in principle integrate in a heritable fashion into the host genome, thus preserving information from the distant evolutionary past [[10]]. Mimivirus EVEs have not been found, and one might suspect that their extraordinarily large genomes mean that they are unlikely to form EVEs. However, we could consider a virophage EVE to exist in the form of Mavericks; in some sense, a mimivirus that donates genes to a subsequently endogenized virophage could be thought of as a ‘vicarious EVE’. This flow of genes, from mimivirus to virophage to host genome, is therefore evident in the amoeba genome.

One proposal to explain the endogenization of virophages is that it could have been positively selected for, since the association with a virophage is beneficial to the host cell (owing to its interference with the replication of the large DNA virus) [[7]]. The survival advantage gained by an integrated virophage could conceivably be the production of virophages as a kind of antiviral response. If the viral threat were lost, then selection for the maintenance of virophage production would be relaxed, explaining the proposed loss of virophage features in Mavericks [[7]]. Because only a handful of virophages have been described, there are insufficient data to investigate the evolutionary dynamics at play. With improvements in sequencing, bioinformatics and metagenomics, viral discovery is increasing exponentially, and moving beyond studies of immediate medical and economic interest. These advances will generate more data that will be suitable for the study of these evolutionary dynamics.

## The entanglement of giant viruses, virophages and their hosts

The discovery of giant viruses has crossed some of the boundaries between viruses and cellular life, although ribosomes remain a distinguishing feature. The conflict between giant viruses and their hosts, with the former also infected by virophages, alongside genomic invasions with related transposons, is reminiscent of Darwin’s tangled bank, recapitulated at the microscopic scale in a droplet of water. Elucidating the role of gene flow between these microscopic entities will reveal their evolutionary dynamics and aspects of the origins of viruses and cellular life.
